# Poster Session II - A308 DIETARY QUALITY, FOOD SECURITY, AND PSYCHOSOCIAL DETERMINANTS IN ADULTS WITH INFLAMMATORY BOWEL DISEASE: INSIGHTS FROM THE MCMASTER IBD NUTRITION REGISTRY

**DOI:** 10.1093/jcag/gwaf042.308

**Published:** 2026-02-13

**Authors:** H Zafar, P Miranda, A Ahmed, R Borojevic, J Blom, M Pinto-Sanchez, D Armstrong

**Affiliations:** Medicine, McMaster University Faculty of Health Sciences, Hamilton, ON, Canada; McMaster University, Hamilton, ON, Canada; McMaster University, Hamilton, ON, Canada; McMaster University, Hamilton, ON, Canada; Hamilton Health Sciences, Hamilton, ON, Canada; McMaster University, Hamilton, ON, Canada; McMaster University, Hamilton, ON, Canada

## Abstract

**Background:**

Diet and nutrition are increasingly recognized as a key modulators of disease activity, symptom burden, and quality of life in Inflammatory Bowel Disease (IBD). Despite this, little is known about the nutrition status, food security, and psychosocial factors influencing dietary behaviours among Canadians living with IBD. Understanding these determinants is critical to inform patient-centred dietary counselling and integrate nutrition care in IBD management.

**Aims:**

To establish a patient registry to characterize dietary quality, diet behaviours, food security, and psychosocial factors among adults with IBD.

**Methods:**

Adults with confirmed IBD attending the McMaster University Digestive Disease outpatient clinic were invited to participate in the IBD nutrition registry. Participants completed standardized electronic surveys on a tablet, prior to their clinic appointment. The registry includes surveys to assess demographics, socio-economic factors, dietary patterns and behaviours, diet quality (Short Healthy Eating Index, sHEI), food security (Canadian Household Food Security Survey), and perceived disease control (IBD-Control-8). Descriptive statistics summarized cohort characteristics, and Spearman’s rank correlations (ρ) examined relationships between sHEI scores and other variables.

**Results:**

A total of 135 patients were enrolled in the registry, with complete data available for 124 participants. Of these, 64.1% were female, with a mean age of 47.3 years, and 64.8% had Crohn’s disease while 35.2% had ulcerative colitis. Food insecurity was reported by 12.8% of participants, slightly below the Canadian average (15.6%). The mean sHEI score was 42.05 ± 10.5 (out of 100), indicating poor overall diet quality (<50) and approximately 16 points below North American population norms (HEI-2015). Higher sHEI scores were significantly correlated with better disease control (ρ = 0.185, p = 0.038). Food avoidance was reported by 61% of participants, and these participants were more likely to report food intolerances (21.3% vs 4.1%, p = 0.016).

**Conclusions:**

Diet quality among individuals with IBD was suboptimal and markedly lower than population norms. Higher diet quality correlated with improved disease control, suggesting a potential protective role of balanced dietary patterns. Food avoidance was highly prevalent among patients with IBD. The McMaster IBD Nutrition Registry provides a scalable framework for the monitoring of nutritional and psychosocial factors in IBD and will inform future interventions to optimize diet-focused clinical care.

**Funding Agencies:**

None

